# Tonic immobility differentiates stress responses in PTSD

**DOI:** 10.1002/brb3.546

**Published:** 2016-09-23

**Authors:** Iro Fragkaki, John Stins, Karin Roelofs, Ruud A. Jongedijk, Muriel A. Hagenaars

**Affiliations:** ^1^ Behavioural Science InstituteRadboud University NijmegenNijmegenThe Netherlands; ^2^ Department of Human Movement SciencesFaculty of Behaviour and Movement SciencesMOVE Research Institute AmsterdamVU University AmsterdamAmsterdamThe Netherlands; ^3^ Behavioural Science Institute and Donders Institute for Brain Cognition and BehaviorRadboud University NijmegenNijmegenThe Netherlands; ^4^ Foundation Centrum '45, Arq Psychotrauma Expert GroupOegstgeestThe Netherlands; ^5^ Department of Clinical PsychologyUtrecht UniversityUtrechtThe Netherlands

**Keywords:** behavioral activation, life events, PTSD, stress, trauma

## Abstract

**Background:**

Tonic immobility (TI) is a state of physical immobility associated with extreme stress and the development of posttraumatic stress disorder (PTSD). However, it is unknown whether TI is associated with a distinct actual stress response, i.e., objective immobility measured by a stabilometric platform. This study made a first step in exploring this as well as differences in body sway responses between PTSD patients and healthy controls. We hypothesized that PTSD would be related to increased body sway under stress, whereas TI would be related to decreased body sway under stress.

**Methods:**

Eye closure was selected as a PTSD‐relevant stress induction procedure. Body sway and heart rate (HR) were measured in 12 PTSD patients and 12 healthy controls in four conditions: (1) maintaining a stable stance with eyes open, (2) with eyes closed, (3) during a mental arithmetic task with eyes open, and (4) with eyes closed.

**Results:**

As predicted, PTSD patients showed increased body sway from eyes open to eyes closed compared to controls and this effect was eliminated by executing the arithmetic task. Most importantly, retrospective self‐reported TI was associated with lower body sway increases in PTSD and higher body sway decreases in controls from eyes‐open to eyes‐closed conditions.

**Conclusions:**

These preliminary findings suggest that eye closure has a different effect on PTSD patients than controls and that high self‐reported TI might indicate a distinct stress response pattern, i.e., a proneness for immobility. It may be relevant to take such individual differences in stress‐response into account in PTSD treatment.

Tonic immobility (TI) is a threat‐related response characterized by physical immobility and muscular rigidity in the face of extreme fear and inescapability (Marx et al., 2008). Although rare in the general population, it is often reported in PTSD patients (23%–37%) (Galliano, Noble, Travis, & Puechl, 1993; Hagenaars, 2016). Peritraumatic TI is a relevant concept in the etiology of PTSD, as it was found to be predictive of PTSD, intrusion development, and poor treatment outcome (Bovin et al., 2008; Fiszman et al., 2008; Hagenaars et al., 2008; Heidt, Marx, & Forsyth, 2005; Lima et al., 2010; Marx et al., 2008). DSM‐5 has recognized that PTSD can present itself in many forms and added a dissociative subtype. Given that TI is associated with distinct post‐trauma behaviors and attitudes (Galliano et al., 1993), PTSD patients with high TI may also form a specific subtype of PTSD, characterized by a distinct behavioral and neurobiological profile.

However, the evidence on TI in humans derived from retrospective self‐report measurements and did not include physiological measurements under experimental manipulations. Only one study experimentally examined body sway with objective measurements (Volchan et al., 2011). Trauma‐exposed subjects with and without PTSD listened to autobiographical scripts while standing on a force platform (Volchan et al., 2011), a device that measures changes in postural position, i.e., objective movement. The authors found that subjective self‐reported TI during the script was associated with reduced body sway and higher heart rate 1 minute postscript. This study nicely demonstrated a relationship between self‐reported TI for a *laboratory stressor* and reduced body sway *after* a stressor, i.e., in the recovery phase. However, the association between self‐reported TI during a *traumatic event* with body sway *during* a stressor still needs to be addressed. This is an important issue as it would verify the validity of self‐report TI measures. Moreover, it would suggest that responding with TI is an automatic (biological or learned) predisposition that will be shown during any encounter with stress or trauma. Several animal studies have corroborated that there is a strong genetic influence on TI [pigs (Erhard & Mendl, 1999); chickens (Gallup, 1974); rats (McGraw & Klemm, 1973)]. In addition, between‐subject trait characteristics have been shown to determine freezing, another threat‐related immobility response (Frank et al., 2006; Hagenaars, Oitzl, & Roelofs, 2014). The choice of the defense strategy [active or passive (immobility)] was also found to be dependent on genetically determined features of serotonin metabolism in the brain (Popova, 2004). Thus, high levels of self‐reported TI during trauma could reflect “TI‐proneness”, i.e., a tendency to a distinct threat response that is expressed when confronted with severe life‐threatening circumstances but also when confronted with moderate stressors. As TI is primarily characterized by reduced movement, TI‐prone individuals should respond with reduced movement during a moderate stressor too.

Therefore, in the present study, we will examine body sway during a general stressor in PTSD patients and healthy controls. PTSD was selected as a clinical population to study TI, because (1) TI is a response to threat and PTSD is by definition related to threatening or traumatic experiences, (2) high levels of TI are prevalent in PTSD patients (23%–37%) (Galliano et al., 1993; Hagenaars, 2016), and (3) TI is predictive of PTSD development (Bovin et al., 2008; Fiszman et al., 2008; Hagenaars et al., 2008; Heidt et al., 2005; Lima et al., 2010; Marx et al., 2008). Furthermore, eye closure was selected as a stressor, because it would interfere with hypervigilance, a specific PTSD symptom. Hypervigilance is a state of continuous monitoring of the environment for potential threat and misinterpretation or excessive reaction to threatening and neutral stimuli (Richards, Benson, Donnelly, & Hadwin, 2014). Many individuals with PTSD, and war veterans in particular, are characterized by increased hypervigilance (Kimble, Fleming, & Bennion, 2013). During eye closure, veterans with PTSD cannot scan the environment, leading to stress due to the uncontrollability of their surroundings. Eye closure also enhances visualization, in general, and PTSD patients report more vivid and emotional memories during eye closure (Hembree, Rauch, & Foa, 2003; Vredeveldt, Hitch, & Baddeley, 2011). Thus, eye closure can be threatening for PTSD patients due to the disrupted scanning of the environment and the anticipation of intrusive images. Moreover, anxiety is related to increased postural sway after sensory manipulations such as eye closure and standing on foam (Stins et al., 2009). A recent study showed that PTSD patients exhibited reduced freezing (i.e., the absence of body sway and heart rate reductions) in response to unpleasant versus neutral and pleasant pictures compared to healthy controls (Fragkaki et al., submitted). Therefore, we expect that PTSD patients will show increased postural instability relative to controls due to increased arousal associated with eye closure.

Second, eye closure during upright standing requires a shift of attention to vestibular and proprioceptive input to maintain balance, which is related to increased body sway or no changes in body sway in healthy individuals (Nieschalk et al., 1999; Prado, Stoffregen, & Duarte, 2007; Stins, Michielsen, Roerdink, & Beek, 2009). This makes it possible to detect a distinct pattern for high TI individuals, i.e., body sway reduction. In contrast, passive viewing of aversive stimuli—an adequate experimental manipulation for inducing analogue threat – is consistently associated with reduced body sway in healthy individuals (Azevedo et al., 2005; Hagenaars, Stins, & Roelofs, 2012; Roelofs, Hagenaars, & Stins, 2010). It thereby disqualifies as a stressor in this study, as decreased body sway in response to unpleasant pictures is the standard response for most individuals in such a paradigm. Performing a cognitive dual task (simultaneously with upright standing) typically leads to a reduction in postural sway as it diverts conscious attention away from balance control and induces a shift to more automatized postural control (Stins et al., 2009). In PTSD patients, a dual task could distract their attention away not only from the balance control, but also from anxiety and hypervigilance, thereby abrogating the effect of eye closure.

Thus, the purpose of this study was to make the first step and investigate (1) body sway responses to a PTSD relevant stressor in PTSD patients and controls, and (2) the association between retrospective trauma‐related TI and body sway responses to that stressor. PTSD patients and controls were assessed under four experimental conditions: (1) maintaining a stable stance with eyes open, (2) with eyes closed, (3) during a mental arithmetic task with eyes open, and (4) with eyes closed. Due to the increased hypervigilance, we hypothesized that PTSD patients would show increased postural sway compared to controls during eye closure. However, we expected that individuals with high self‐report TI would not show this increase, but rather they would exhibit reduced body sway during eye closure, i.e., greater postural immobility. Finally, due to the attentional distracting effect of the cognitive task, we expected the differences between PTSD patients and controls to be eliminated during the task conditions. We measured heart rate as a manipulation check, expecting higher heart rate during the execution of a mental arithmetic task (Orr, Meyerhoff, Edwards, & Pitnam, 1998; Skoluda et al., 2015). We also explored the association between TI and heart rate during eye closure, as their relation is still unknown during a stressor in humans (Hagenaars et al., 2014).

## 1 Method

### 1.1 Participants

The study included 12 male veterans with PTSD recruited from the Foundation Centrum ‘45, the Dutch national center specialized in the treatment of complex trauma victims, and a comparison group of 12 healthy males. The heart rate (HR) data were missing for one participant with PTSD so he was excluded from the analysis. Mean age in the total sample was 43.96 (*SD* = 8.27). The participants were informed about the procedure of the study, gave written informed consent, and received financial compensation of 15 euro. The study was conducted in accordance with the Declaration of Helsinki and approved by the Ethics Committee of Leiden University Medical Center.

### 1.2 Material

#### 1.2.1 Body sway

Postural movements were measured with a custom made 1 × 1 stabilometric platform at a sample frequency of 100 Hz. The length of the sway path (sway path length; SPL) of the center of pressure was used as an outcome measure, which is the total of the center of pressure in the anterior‐posterior and mediolateral plane over the measurement interval. Low values are indicative of the near absence of bodily motion, whereas high values indicate increased bodily movement (and eventually loss of postural stability).

#### 1.2.2 Heart rate

Heart rate (HR) was recorded with a polar band (Heart Rate Telemetry Systems). The polar band was placed at the height of the sternum and the signal was transformed to beats per minute.

#### 1.2.3 Tonic immobility

We administered the Tonic Immobility Scale—Adult Form (TIS‐A; Forsyth et al., 2000) to measure subjective retrospective TI. We used an adapted form that referred to any trauma type instead of sexual trauma only (see also Taylor, Stapleton, & Asmundson, 2007). TIS includes 10 self‐report questions that assess peritraumatic tonic immobility (TIS‐TI) and peritraumatic fear (TIS‐Fear) responses rated on a 7‐point Likert scale (0–6). In this experiment, we used the 7‐item TIS‐TI, which has an excellent internal consistency (Cronbach's α = .94; Fusé et al., 2007). Participants were asked to complete this measurement referring to their worst traumatic experience.

#### 1.2.4 PTSD diagnostic status

PTSD symptoms were assessed with the Clinician‐Administered PTSD Scale (CAPS‐IV; Blake et al., 1990). CAPS‐IV is a 30‐item structured interview that assesses the frequency and intensity of PTSD symptoms. The items are rated on a 5‐point scale (0–4) for frequency and intensity of symptoms respectively that can be summed to a 9‐point scale (0–8) score for each symptom. Participants are asked to answer the questions referring to their worst traumatic experience. CAPS‐IV is a widely used instrument for PTSD with established psychometric properties (Weathers, Keane, & Davidson, 2001).

### 1.3 Procedure

Firstly, participants completed the TIS‐A to measure retrospective TI on their worst traumatic experience as identified during the CAPS‐IV. During the experimental phase, the participants entered a dimly lit room and they were asked to step onto a stabilometric platform, keep their feet at the same position in a slightly splayed stance, and look at the monitor with the fixation cross displayed in front of them (eye level; distance 1 m). The experimental phase included four conditions: (1) standing still with eyes open looking at a fixation cross while holding their arms alongside their body (EO‐NoTask), (2) standing still with eyes closed (EC‐NoTask), (3), standing with eyes open while performing a mental arithmetic task of counting backwards in steps of 7 from a starting value around 300 (EO‐Task), and (4) standing with eyes closed while performing the same task (EC‐Task). Each condition lasted 30 s and was conducted four times. The 16 trials were presented in random order and the total duration of balance recordings was 8 min.

### 1.4 Data analyses

Postural sway excursions were analyzed with MATLAB and further statistical analyses were performed with the Statistical Package for Social Sciences (IBM SPSS 20.0). The time‐series were filtered (second‐order low‐pass Butterworth filter, cut‐off frequency 10 Hz), and the posturographic data (SPL) and HR data were averaged over the four trials of each condition. The data were analyzed with 2 × 2 × 2 mixed analyses of variance (ANOVA) with within‐subject factors Vision (Eyes Open, Eyes Closed) and Task (No‐Task, Task), between‐subject factor Group (PTSD patients, Controls) and TI as a covariate. Separate two‐way ANOVAs were performed to examine the direction of the effects in three‐way interactions. The alpha level for all the analyses was .05 and the effect sizes are presented as partial eta‐squared ($\eta_{p}^{2}$).

## 2 Results

### 2.1 Descriptives

**Figure 1:**
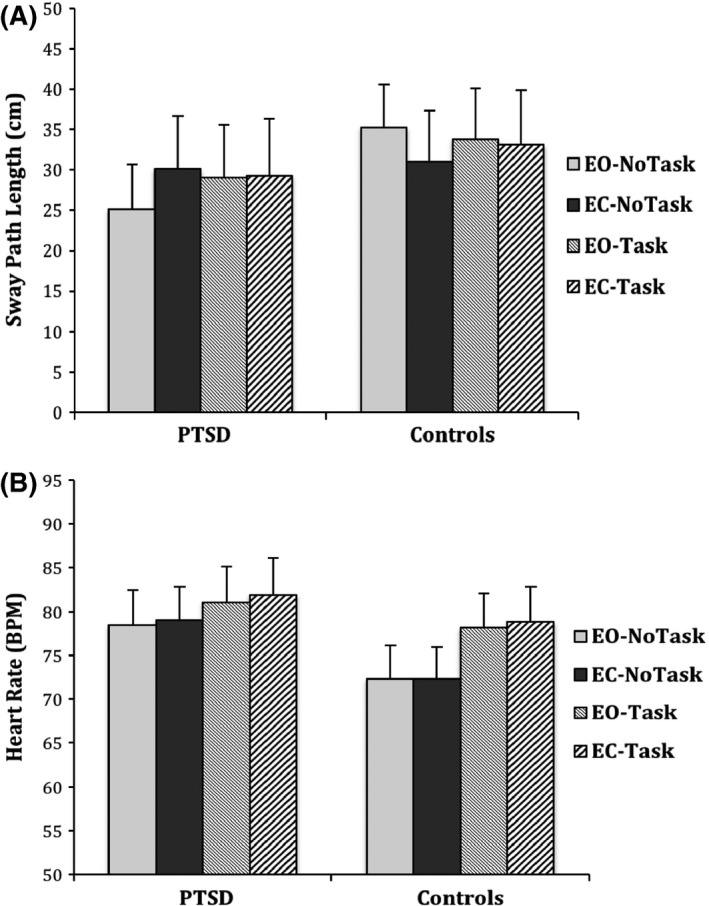
Mean of sway path length (SPL, panel (A) and heart rate (HR, panel (B) for PTSD group, and Controls for the four conditions. Error bars denote standard errors of mean

Participants in the PTSD group had a mean age of 39.82 (*SD* = 6.75), which was significantly lower than the control group (*M* = 47.75, *SD* = 7.92), *t*(20) = −2.591, *p *=* *.017. Participants in the PTSD group were more likely to have lower education and be unemployed (*p*s < .05). The mean score on the CAPS was 86.92 (*SD* = 13.64, 95% CI [78.25, 95.58]) for PTSD patients, which indicates severe PTSD (Weathers et al., 2001). The mean score for TI was 15.83 (*SD* = 9.16) (PTSD: *M* = 20.73, *SD* = 10.61; Controls: *M *=* *11.33, *SD* = 4.40). Mann–Whitney test showed that PTSD patients (Mdn = 15.05) had significantly higher TI scores than controls (Mdn = 9.21) (*U* = 32.500, *z* = −2.066, *p *=* *.039). Figure 1 depicts the raw mean and standard error for SPL and HR in all conditions for both groups.

### 2.2 Posturography

There was a significant three‐way Vision × Task × Group interaction, *F*(1, 20) = 9.330, *p *=* *.006, $\eta_{p}^{2}$ = .318. Separate ANOVAs with and without the task and for each group showed that without the task SPL increased for the PTSD group, but decreased for Controls, *F*(1, 20) = 33.563, *p *<* *.001, $\eta_{p}^{2}$ = .627. In contrast, during execution of the cognitive task, this effect was no longer significant, *F*(1, 20) = .459, *p *=* *.506.

The Vision x Group interaction was significant, *F*(1, 20) = 13.977, *p *=* *.001, $\eta_{p}^{2}$ = .411, indicating distinct responses to eye closure for PTSD patients and Controls. The Vision x Task interaction was also significant, *F*(1, 20) = 5.939, *p *=* *.024, $\eta_{p}^{2}$ = .229, suggesting that SPL increased from EO to EC in No‐Task, but not in Task. The Group × Task interaction was not significant (*F *<* *.376, *p *>* *.546). Finally, there was a significant main effect of Vision, *F*(1, 20) = 10.498, *p *=* *.004, $\eta_{p}^{2}$ = .344, but not for Group or Task (both *F*s < .505, both *p*s > .486). SPL was significantly higher in eyes closed than in eyes open conditions across groups.

**Figure 2:**
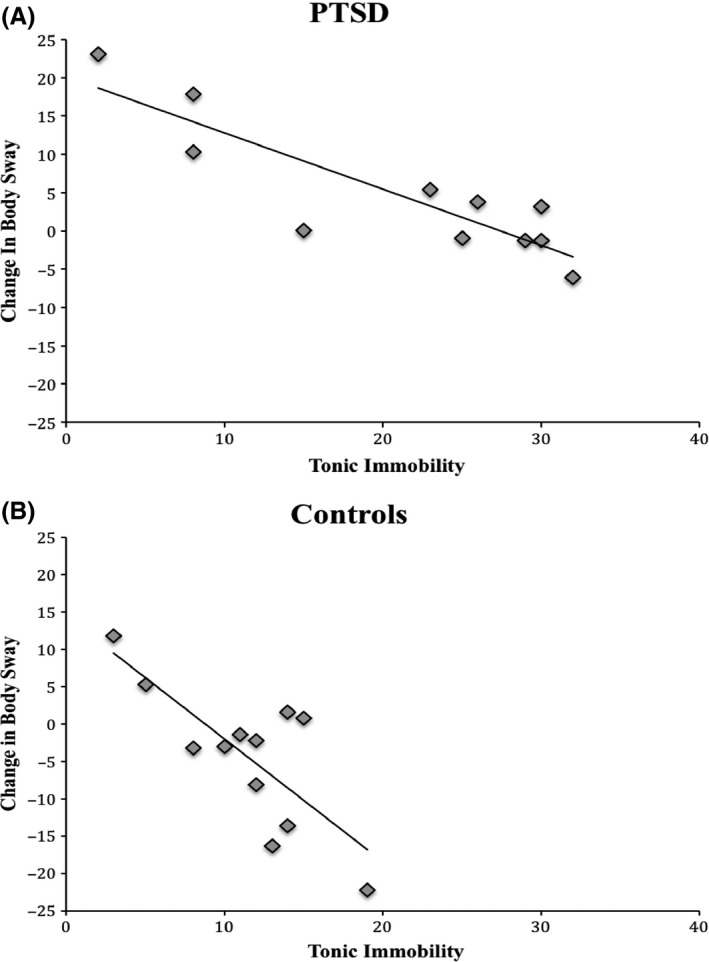
Scatterplot with regression line presenting the correlation between tonic immobility and change in body sway (sway path length; in centimeters) when participants had their eyes closed compared to eyes open for PTSD patients (panel A) and Controls (panel B). Positive values scores indicate increases and negative values indicate decreases in body sway from Eyes‐Open to Eyes‐Closed

With respect to TI, the three‐way Vision × Task × TI interaction was significant, *F*(1, 20) = 6.588, *p *=* *.018, $\eta_{p}^{2}$ = .248. Post hoc partial correlations controlling for Group showed that TI was correlated with decreases in SPL from EO to EC in No‐Task (*r* = −.76, *p *<* *.001; see Fig. 2), but not during the execution of the task (*r *=* *.19, *p *=* *.41). TI was not related to changes in SPL for Task versus No‐Task with EO (*r *=* *.33, *p *=* *.12) or with EC (*r *=* *.17, *p *=* *.43). The two‐way Vision × TI interaction was also significant, *F*(1, 20) = 12.687, *p *=* *.002, $\eta_{p}^{2}$ = .388, but not the Task × TI interaction, *F*(1, 20) = .219, *p *=* *.645. Partial correlations controlling for Group showed that TI was correlated with decreases in SPL from EO to EC conditions (*r *=* *−.623, *p *=* *.002).

### 2.3 Heart rate

There were no interaction effects for Group, Vision, Task, and TI (all *F*s < 2.30, all *p*s > .14). Three main effects were significant though: Group (*F*[1, 20] = 4.372, *p *=* *.050, $\eta_{p}^{2}$ = .179), Task (*F*[1, 20] = 9.866, *p *=* *.005, $\eta_{p}^{2}$ = .330), and TI (*F*[1, 20] = 5.997, *p *=* *.024, $\eta_{p}^{2}$ = .231). Specifically, PTSD patients had a higher overall HR than Controls, HR increased from NoTask to Task, and higher TI was related to lower HR controlling for Group (*r *=* *−.48, *p *=* *.024). The main effect of Vision was not significant, *F*(1, 20) = 1.043, *p *=* *.319.

## 3 Discussion

The aim of this study was to make a first step in exploring differences in body sway responses to stress between PTSD and controls and the association between self‐report TI and actual body sway in response to a PTSD‐relevant stressor. As expected, eye closure resulted in increased body sway for PTSD patients, but not for controls. Directing attention inwards might have elicited a sense of uncontrollability because of reduced visual input, possibly resulting in fear or restlessness. With eyes closed, PTSD patients are prevented from scanning the environment for threatening cues and/or they could temporarily experience or anticipate the occurrence of intrusive memories. Veterans may be especially sensitive to reduced visual control, because they were used to operate in a hostile environment with danger everywhere and were trained to be alert to threat. Speculatively, increased body sway could be an indicator of elevated fight‐flight behavior in PTSD in response to eye closure. This is in line with other findings supporting a lack of freezing response in PTSD patients (Adenauer et al., 2010). This effect was observed only without the task, suggesting that the task indeed functioned as a distractor for PTSD patients. During the task, their attention might have shifted away from scanning the environment or inhibiting intrusive memories, resulting in a decrease in anxiety.

Most importantly, confirming our hypothesis, subjective, retrospective TI was associated with decreases in body sway during eye closure without the task in the whole sample. This finding is striking as it reveals a distinct response to stress in individuals with high TI. Remarkably, high TI was associated with reduced body sway whereas PTSD was associated with increased body sway in response to eye closure. More specifically, PTSD patients with high TI exhibited a reduced increase in body sway from eyes‐open to eyes‐closed, whereas Controls with high TI exhibited greater decreases in body sway, both possibly indicating a distinct threat response that is characterized by a more prominent role of the parasympathetic nervous system. The presence of a distinct response to threat in TI‐prone individuals warrants further exploration in future research. It will be interesting for future studies to investigate these patterns in a larger sample size of PTSD patients with high versus low TI to shed more light on this issue.

PTSD is a multi‐faceted disorder, which was recognized by including additional symptom presentations in DSM‐5 (American Psychiatric Association, 2013; Galatzer‐Levy & Bryant, 2013). Our data, as well as previous studies reporting decreased treatment response in PTSD with TI (Fiszman et al., 2008; Lima et al., 2010), might indicate a TI‐specific PTSD subtype. This may be highly relevant, as they possibly suggest that treatments should be adjusted to this PTSD‐type. Indeed, PTSD patients with high levels of TI responded poorly to pharmacological treatment (Fiszman et al., 2008; Lima et al., 2010). Popova (2004) suggested that the choice of a defense strategy is based on serotonin metabolism in the brain and Lima et al. (2010) argued that PTSD patients that respond with TI might have higher levels of serotonin and reduced sensitivity of postsynaptic serotonin receptors, which might explain the ineffectiveness of antidepressants in these individuals. Treatments can be adjusted in several ways. First, TI induces feelings of shame and guilt (Bovin et al., 2014) so psychoeducation on the automatic nature of this response may be especially important in these specific PTSD patients, as it was already suggested 35 years ago (Bovin et al., 2014; Suarez & Jr Gallup, 1979). Also, although exposure treatments are generally effective for PTSD, the effects are stronger for fear and anxiety than for other emotions and cognitions (Schnyder & Cloitre, 2015). Emotions/cognitions such as shame and guilt may merit additional treatment strategies focusing on other emotional responses. For example, imagery rescripting was equally effective for fear‐related emotions but more effective in reducing other emotional responses (such as anger; Arntz, Tiesema, & Kindt, 2007). Imagery rescripting techniques might also be used to address peritraumatic TI responses and related emotions. Overall, our data, as well as previous studies reporting an association between high TI and PTSD severity or poor treatment response, might indicate a TI‐specific PTSD form which could be in need of more tailored interventions.

With respect to heart rate, we found the expected heart rate increase during a cognitive task, which is in line with previous findings (Orr et al., 1998; Skoluda et al., 2015). The task requires a (mental) effort, which is associated with increased sympathetic activity (Orr et al., 1998; Skoluda et al., 2015). PTSD patients had overall higher heart rates than controls, consistent with previous studies that found overall increased heart rate in PTSD, suggesting a lack of de‐activation at rest (Buckley & Kaloupek, 2001; Pole, 2007). TI was related to lower heart rate. This seems contradictory to the two human studies reporting that TI was related to heart rate increases in response to trauma‐relevant stimuli (Alves et al., 2014; Volchan et al., 2011). However, our effect was general and not condition specific, and thus not related to the stressor. Heart rate is affected by both the sympathetic and parasympathetic nervous system activity and thus highly variable, which may result in seemingly contradictory findings. Reduced heart rate could be related to freezing‐like behavior, or nonspecific attentive processing during the whole experiment. Previous research in animals presented both increases and decreases in heart rate during TI induction (Hagenaars et al., 2014; Nash, Gallup, & Czech, 1976). Again, relative sympathetic and parasympathetic activity changes over time during TI, and thus a simple mean heart rate may not cover the complex of physiological changes during TI over time (Carli, 1974). Future research might include skin conductance in addition to heart rate in order to gain more information on pure arousal. Interestingly, eye closure may be a TI‐congruent stressor as it is accompanied by intermittent eye closure in animals. Moreover, occurrence of eye closure during TI is associated with longer TI duration in animals (Gallup, Nash, & Wagner, 1971).

Several limitations should also be mentioned. Most importantly, the sample size was small and the control group did not include war veterans thus replication studies are needed. Subjective measures of fear and TI between the conditions could also provide important information, as well as assessment of startle, skin conductance, and body temperature. Previous studies found hypothermia during TI induction in animals (Eddy & Gallup, 1990; Nash et al., 1976) and it would be interesting to test whether similar temperature changes can be found in high and low TI PTSD patients. Moreover, a baseline measure of heart rate could have given us a clearer picture of heart rate (re)activity. Finally, we did not examine the participants' performance on the task, which could have provided further information about attentional and memory processes and the trade‐off between behavioral responses and task performance.

Overall, this study contributes to the knowledge on automatic responses to threat in PTSD and revealed preliminary evidence on a distinctive pattern in individuals with higher TI in response to experimentally induced stress. That is, eye closure leads to increased postural sway in the PTSD group, but PTSD patients with high TI exhibited a lower increase in body sway, and Controls exhibited a greater decrease in body sway. This distinctive response raises questions about other potential differences in PTSD based on levels of TI that warrant future exploration. Additionally, these findings suggest that traumatic experiences and the associated TI may become embodied in postural control systems. Future research is highly needed to corroborate these findings in larger samples of PTSD patients and compare PTSD with other threat‐related disorders and anxiety disorders to establish whether this association is a transdiagnostic symptom or specific to PTSD.

## Conflict of Interest

There is no conflict of interest.

## Funding Information

Netherlands Organisation for Scientific Research (NWO) (Grant/Award Number: VENI Grant/#451‐09‐018, VIDI Grant/#452‐07‐08).
